# Interference chromatography: a novel approach to optimizing chromatographic selectivity and separation performance for virus purification

**DOI:** 10.1186/s12896-020-00627-w

**Published:** 2020-06-17

**Authors:** Lisa A. Santry, Renaud Jacquemart, Melissa Vandersluis, Mochao Zhao, Jake M. Domm, Thomas M. McAusland, Xiaojiao Shang, Pierre M. Major, James G. Stout, Sarah K. Wootton

**Affiliations:** 1 Department of Pathobiology, University of Guelph, Guelph, Ontario N1G 2W1 Canada; 2 MilliporeSigma, 5295 John Lucas Drive, Burlington, Ontario L7L 6A8 Canada; 3 Present Address: BioVectra Inc., 24 Ivey Lane, PO Box 766, Windsor, Nova Scotia B0N 2T0 Canada; 4 Juravinski Cancer Centre, 699 Concession Street, Hamilton, ON L8V 5C2 Canada

**Keywords:** Interference chromatography, Anion exchange membrane, Single-use bioprocessing, Oncolytic virotherapy, Newcastle disease virus, Virus purification

## Abstract

**Background:**

Oncolytic viruses are playing an increasingly important role in cancer immunotherapy applications. Given the preclinical and clinical efficacy of these virus-based therapeutics, there is a need for fast, simple, and inexpensive downstream processing methodologies to purify biologically active viral agents that meet the increasingly higher safety standards stipulated by regulatory authorities like the Food and Drug Administration and the European Agency for the Evaluation of Medicinal Products. However, the production of virus materials for clinical dosing of oncolytic virotherapies is currently limited—in quantity, quality, and timeliness—by current purification technologies. Adsorption of virus particles to solid phases provides a convenient and practical choice for large-scale fractionation and recovery of viruses from cell and media contaminants. Indeed, chromatography has been deemed the most promising technology for large-scale purification of viruses for biomedical applications. The implementation of new chromatography media has improved process performance, but low yields and long processing times required to reach the desired purity are still limiting.

**Results:**

Here we report the development of an interference chromatography-based process for purifying high titer, clinical grade oncolytic Newcastle disease virus using NatriFlo® HD-Q membrane technology. This novel approach to optimizing chromatographic performance utilizes differences in molecular bonding interactions to achieve high purity in a single ion exchange step.

**Conclusions:**

When used in conjunction with membrane chromatography, this high yield method based on interference chromatography has the potential to deliver efficient, scalable processes to enable viable production of oncolytic virotherapies.

## Background

A major challenge in virus production for gene and oncolytic virotherapies is the time-efficient purification of large quantities of clinical grade material [1, 2]. In vaccine manufacturing, limitations in virus purification such as low productivity, high capital expenditure, scalability challenges, and low overall process yields are already experienced with conventional technologies including ultracentrifugation, tangential flow filtration (TFF), size exclusion chromatography (also known as gel filtration), and DNase treatments [3]. These limitations are magnified in the production of gene and oncolytic virotherapies due to even higher product quality requirements which must meet a variety of specific criteria for purity (endotoxin, contaminating host cell DNA), potency, identity (endpoint PCR, sequencing, restriction analysis), stability, and product characterization (e.g. ratio of total virus particles to infectious particles) prior to their use in the clinic [4]. Moreover, there is a need for much larger quantities of viruses to treat patients, particularly for oncolytic viruses (OVs), many of which are administered intravenously and therefore must meet strict purity standards. This often needs to be achieved in a short period of time and sometimes in a small footprint facility located at point-of-care.

Thanks to its improved productivity, reproducibility, cost, and yield, chromatography methods have gained in popularity and have been developed for many commonly-used viral vectors [5], including adenovirus [6–9], adeno-associated virus [10, 11], and lentivirus vectors [12–14], as well as oncolytic viruses such as reovirus [9], herpesvirus [15, 16] and vaccinia virus [17, 18]. These chromatography techniques can be broadly classified into affinity chromatography, ion-exchange chromatography, hydrophobic interaction chromatography, and size-exclusion chromatography. Affinity chromatography methods exploit the natural ability of some viruses to bind cell surface molecules such as heparin or sialic acids, or make use of antibodies to enrich specific viruses and lead to high product purity in a single step thereby reducing the number of downstream process (DSP) operations [19]. Ionic exchange resins can selectively enrich for viruses based on unique net surface charge. Ion exchange (IEX) chromatography has gained in popularity throughout the OV community (IEX is used for T-Vec (Imlygic®) and Reolysin® [20]), but the strategy can still be improved to deliver purification schemes compatible with the needs of these new types of viral therapies. For instance, the implementation of technologies such as membrane chromatography or monoliths has improved the productivity and cost profiles when compared to IEX resins [21, 22]. Hydrophobic interaction chromatography separates biomolecules based on their degree of surface hydrophobicity and has been used to purify a wide range of viruses including foot-and-mouth disease virus, influenza virus and vaccinia virus [17, 23–25]. Finally, given the large size of viruses compared to most cellular factors, size exclusion chromatography is frequently applied as a singular- or complementary step during industrial virus purification [26, 27]. Chromatography separation methods therefore offer a growing alternative to gradient ultracentrifugation for producing high purity virus in an industrial setting.

In this paper, we present interference chromatography, a novel approach to optimizing chromatographic selectivity and separation performance for virus production. Interference chromatography involves the addition of interfering agents to the sample and mobile phase that will modify the molecular interactions between the sample and chromatographic matrix. Altering these interactions can improve the selectivity, purification, and throughput of the target molecule separation.

Newcastle disease virus (NDV) was chosen as the model virus for this proof of concept study because of its potent anti-neoplastic properties [28, 29]. The observed antitumor effect of NDV is attributed not only to its tumor-selective replication leading to significant oncolytic activity [30–33], but also to its potent immune stimulatory properties [29, 34–36], which together results in tumor regression and systemic tumor-specific immunity [37–39]. Additionally, NDV has the longest history of use in clinical applications (> 50 years) of any OV, with reportedly low side effects and a high safety profile [40]. The absence of recombination (genomic stability), its exclusive cytoplasmic replication cycle (inability to integrate into host cell genome), and the mild side effects experienced by cancer patients further promote NDV as an attractive virotherapeutic agent.

Given the myriad of promising pre-clinical and human clinical data demonstrating the efficacy of NDV as an oncolytic immunostimulatory agent [41–44], scalable manufacturing methods for purifying NDV for the purpose of human clinical testing are greatly needed. Stocks of the virus are prepared by growth in either specific pathogen free embryonated chicken eggs or in cell culture; however, growth in embryonated eggs is currently the only method for producing high titer NDV stocks (average titers ranging from 10^7^ to 10^8^ TCID_50_/mL), as to date, no cell lines able to produce comparable titers of NDV have been identified [45]. There are a number of challenges associated with clinical grade production of NDV including the removal of host cell proteins and genetic material from the allantoic fluid, variation in NDV particle size and morphology [46, 47], susceptibility of the virus to shearing [48], and the requirement for upwards of 10^13^ PFU per patient [41] (a total of 10^14^ PFU would be required for a Phase I-II trial) to be delivered intravenously. Current virus purification processes employ multiple steps [45, 49], which are typically centrifugation-based; leading to slow, labor-intensive processes with low yields and high failure rates in a manufacturing environment, highlighting the need for new purification strategies that deliver ultra-pure products, with short development and production times and at low manufacturing costs. Here we describe an optimized, scalable interference chromatography method for the purification of high titer, ultra clean NDV from allantoic fluid.

## Results

### Screening interference agents

**Fig. 1:**
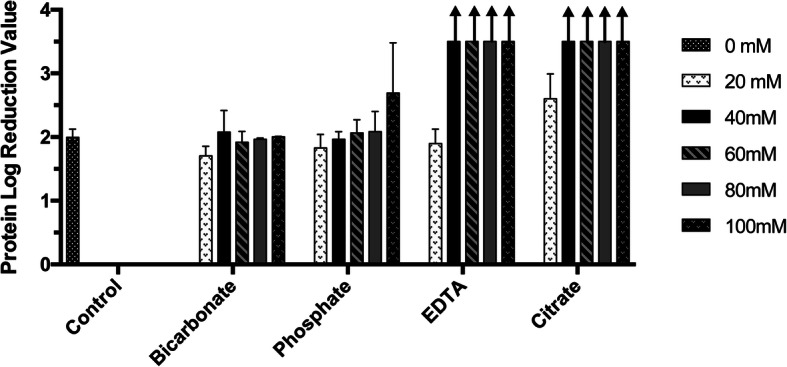
Screening of interference agents (bicarbonate, phosphate, EDTA, and citrate) and interference agent concentrations (20 mM, 40 mM, 60 mM, 80 mM, and 100 mM) in AEX virus purification and their effect on protein clearance. The control represents the normal chromatography mode without addition of interference agents. Protein in the elution was non-detectable for experiments with 100 mM phosphate, 40 mM, 60 mM, 80 mM, and 100 mM EDTA, as well as 60 mM, 80 mM, and 100 mM citrate, therefore the calculation for log reduction value results in an infinite number (depicted by upward arrows). (*n* = 2 for bicarbonate, phosphate, and EDTA experiments, *n* = 3 for the control and citrate experiments, error bars show mean ± SD)

**Fig. 2:**
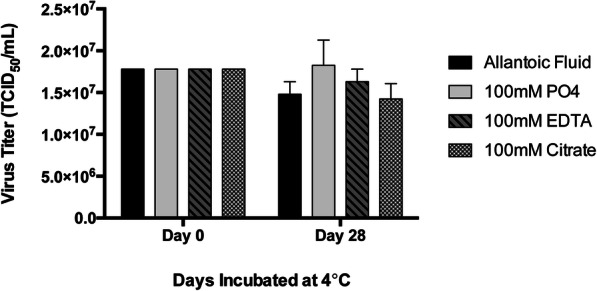
Stability of NDV in the interference buffers. NDV was incubated in 100 mM PO4, 100 mM EDTA and 100 mM citrate for 28 days at 4 °C and then titered (*n* = 3). NDV appeared to be stable in all three interference buffers as there was no statistically significant decrease in viability compared to the control. Mean ± SD are shown

For optimization of different interference conditions, one layer of NatriFlo® HD-Q membrane (membrane volume = 0.1 mL) assembled in a 25 mm diameter stainless steel housing (25 mm SS device) was used. The interference chromatography method introduces a significant boost in host cell protein (HCP) removal when purifying NDV with anion exchange media. As illustrated in Fig. 1, the conventional technique with no interference agents (control), achieved a protein log reduction value (LRV) of 1.99. When each of the four different interference chromatography agents (bicarbonate, phosphate, EDTA and citrate) were added separately to the equilibration buffer and the NDV sample (allanotic fluid), protein removal was dramatically improved in the cases of EDTA and citrate, and small improvements in purity were observed with phosphate. As the concentration of those interference agents is increased, the product purity is improved. Bicarbonate does not appear to significantly improve protein clearance with this particular feed. In this proof of concept with NDV in allantoic fluid, interference with citrate provided the best protein clearance (2.6 LRV of total protein with only 20 mM citrate) followed by EDTA, phosphate, and then bicarbonate (Fig. 1). To confirm that the interference agents did not compromise virus infectivity, the stability of NDV in 100 mM phosphate, 100 mM EDTA and 100 mM citrate buffers was evaluated. As shown in Fig. 2, NDV was stable for 28 days in all three buffers when stored at 4 °C.

**Fig. 3:**
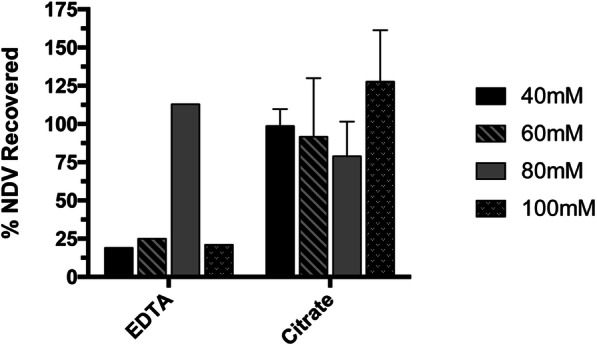
Percentage of NDV recovered in the elution for interference conditions that resulted in protein clearance down to non-detectable levels. Recovery was measured using TCID_50_ assay. Recovery for the 80 mM EDTA experiment is likely an outlier. (*n* = 1 for EDTA experiments, *n* = 3 for citrate experiments, error bars show mean ± SD)

As EDTA and citrate buffer were the top two performing interference agents in terms of protein clearance, resulting in non-detectable amounts of protein in the elution, they were compared to assess the effect of interference on the virus recovery. Figure 3 shows the percentage of NDV loaded onto the membrane that was recovered in the elution. Experiments with citrate buffers generally have high recoveries (> 80%) while EDTA experiments have poor recoveries (< 50%) except in the case of 80 mM EDTA, which appears to be an outlier.

### Conductivity control

**Fig. 4:**
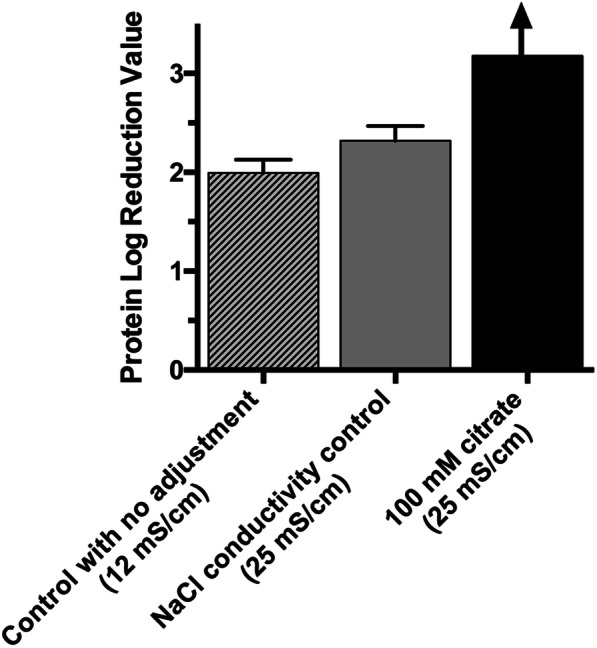
Control study to evaluate whether interference properties are caused by the conductivity of the solution or the unique characteristics of the interference agent. NDV sample with 100 mM citrate enables better clearance than NDV sample with the same conductivity (adjusted by sodium chloride). No protein was detected in the elution for 100 mM citrate, therefore the calculation for log reduction value results in an infinite number (depicted by upward arrow). (*n* = 2 for control with no adjustment, *n* = 3 for conductivity control and 100 mM citrate, mean values ± SD are shown)

The aim of the conductivity control test is to determine if it is the conductivity of the solution or specific properties of the interference agent that improve the impurity clearance. The conductivity control feed, prepared with sodium chloride, is adjusted to the same conductivity as the feed containing 100 mM citrate (25 mS/cm). Figure 4 shows that while increasing the conductivity does improve impurity reduction performance as compared to a feed with no conductivity adjustment, the feed containing 100 mM citrate enables much greater product purity.

### Capacity test with optimal interference condition

**Fig. 5:**
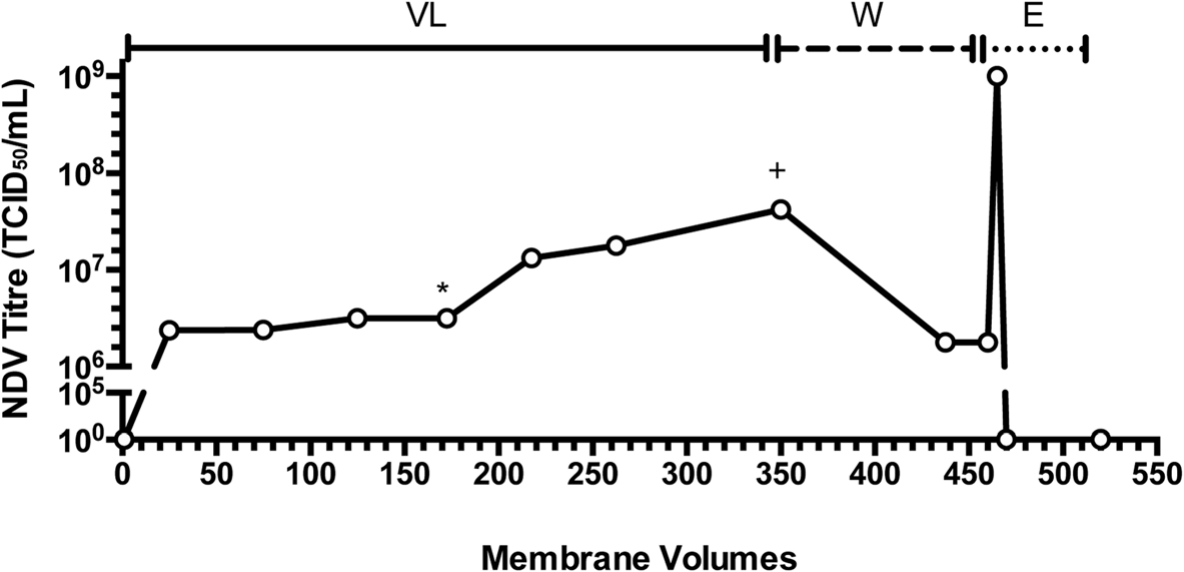
Capacity test with optimal interference condition of 100 mM citrate buffer. Under the interference condition of 100 mM citrate, the sample load was increased to determine the NDV loading capacity. NDV containing allantoic fluid (4.22 × 10^7^ TCID50/mL) was loaded onto the column (VL). The membrane reached capacity at approximately 170 membrane volumes (MV) (*), where an increase in virus was observed in the flow through. Breakthrough continued until approximately 350 MV (+), where the virus in the flow through was similar to the concentration of input virus. At this point, a wash step (W) was applied to the membrane, followed by an elution step (E) at 460 MV. The reconstructed chromatogram was created from offline measurements of NDV titres from fractions taken throughout the experiment

Under the interference condition of 100 mM citrate, the sample load was increased to determine the NDV loading capacity. Citrate was chosen as the interference agent because it proved to be most effective at enhancing impurity reduction, even at low concentrations, while still providing high virus recovery. For these experiments, which were conducted in triplicate, the 25 mm SS device with a membrane volume of 0.1 mL was used. A representative chromatogram is shown in Fig. 5, in which NDV at a concentration of 4.22 × 10^7^ TCID_50_/mL was loaded onto the membrane. Virus was titered in the flow through at different intervals. Membrane volumes were calculated by dividing the liquid volume (in mL) by the membrane volume of the 25 mm SS device used (0.1 mL). The membrane reached loading capacity around 170 membrane volumes, where an increase in virus was detected in the flow through. This continued to increase until 350 MV was reached, where virus in the flow through equated to the concentration of input virus. At this point, a wash step was applied to the membrane, followed by an elution step at 460 MV. A total virus load of 2.1 × 10^10^ TCID50/mL of membrane exceeded the capacity of the membrane resulting in virus break through during loading. Based on the amount of NDV collected in the elution, the binding capacities for the three independent experiments were 1.5 × 10^10^, 1.0 × 10^10^, and 4.2 × 10^9^ TCID_50_/mL of membrane. Protein clearance was greater than 99.9% for all three capacity tests, with 17 μg, 21 μg, and 11 μg total protein in the elution, respectively. This corresponds to a ratio of NDV to protein of 1.1 × 10^8^ TCID_50_/μg, 2.1 × 10^8^ TCID_50_/μg, and 2.7 × 10^8^ TCID_50_/μg. DNA clearance was also measured and results showed reductions of 96, 51, and 57% of DNA for each of the three capacity tests.

### Scalability

**Fig. 6:**
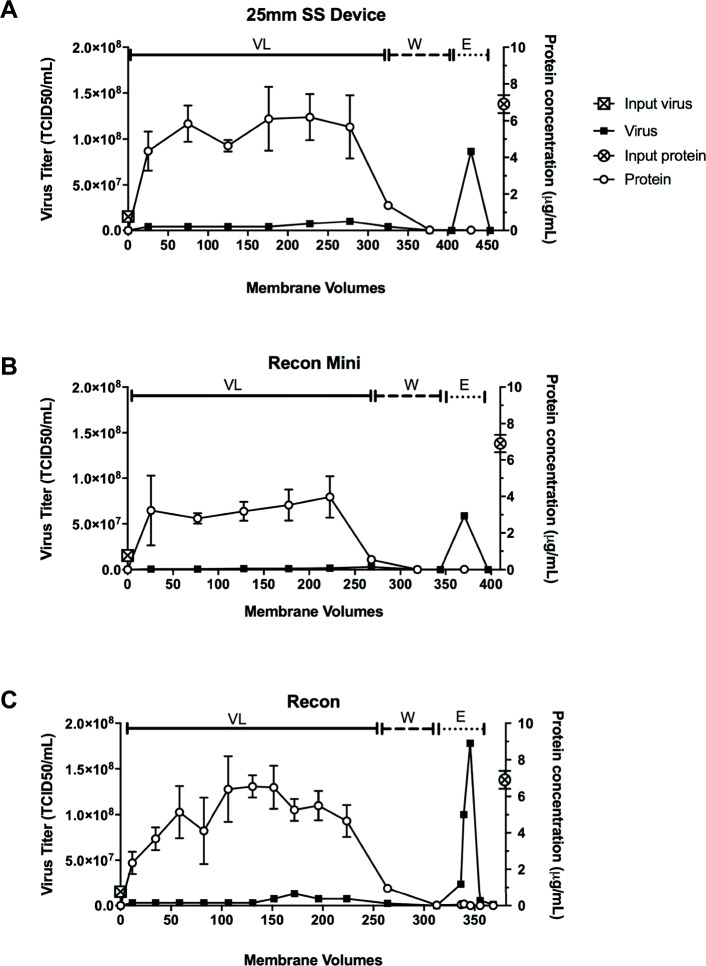
The 25 mm SS device (0.1 mL membrane volume) (**a**), the NatriFlo® HD-Q Recon Mini (0.2 mL membrane volume) (**b**) and the NatriFlo® HD-Q Recon (0.8 mL membrane volume) (**c**) were loaded with allantoic fluid containing NDV (VL) followed by two wash steps (W) and an elution step (E) and binding kinetics evaluated with increasing membrane volumes. The amount of infectious virus and protein in the eluate was quantified over time by TCID_50_ assay and Bradford protein assay, respectively. Runs were repeated three times. Proteins were analyzed in triplicate, virus samples (*n* = 3) were pooled and titered. Means ±SD are shown

To evaluate scalability, devices with increasingly larger membrane volumes were tested. The 25 mm SS device (0.1 mL membrane volume), the NatriFlo® HD-Q Recon Mini (0.2 mL membrane volume) and the NatriFlo® HD-Q Recon (0.8 mL membrane volume) were loaded with 30 mL, 50 mL and 200 mL of allantoic fluid, respectively, and binding kinetics evaluated (Fig. 6). Percent virus recovery was 90, 68 and 86% for the 25 mm SS device, the NatriFlo® HD-Q Recon Mini and the NatriFlo® HD-Q Recon, respectively, with total protein clearance of > 99.9% and total DNA clearance of ~ 55% for all three devices. Taken together, these data demonstrate the scalability of NatriFlo® HD-Q membrane technology for virus purification.

### Reproducibility and purification performance

**Fig. 7:**
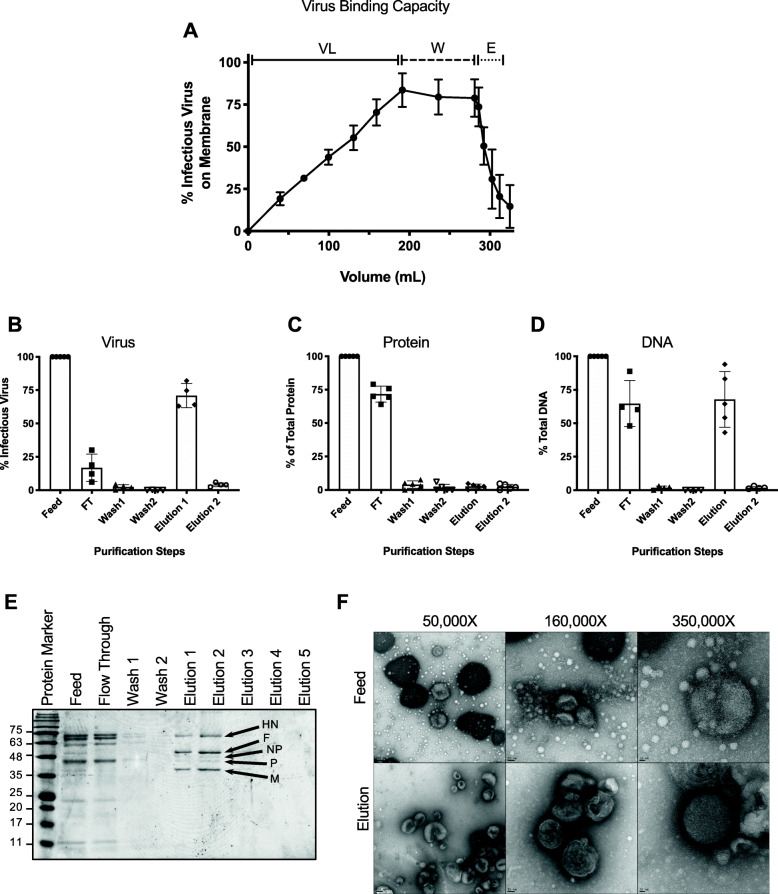
Virus binding kinetics and analysis of large-scale production runs. A total of 200 mL of allantoic fluid (VL) was loaded onto the NatriFlo® HD-Q Recon A membrane followed by two wash steps (W) of 45 mL each and a 30 mL elution step. **a** Virus binding kinetics showing average amount of infectious virus bound to the membrane throughout the three purification runs.. The experiment was conducted on three separate occasions using three different batches of feed. Percent infectious virus (**b**), protein (**c**) and DNA (**d**) in the feed, flow through (FT), wash and elution fractions (*n* = 4–5, means ±SD are shown). **e** Representative SDS-PAGE analysis of samples (10 μL) taken from the feed, flow through, wash and elution steps. Proteins were separated on a 12% Tris-glycine polyacrylamide gel and stained with Coomassie blue. The elution fraction appeared to be enriched for viral proteins HN (Hemagglutinin Neuraminidase; 62.8 kDa), F (Fusion protein; 59 kDa), NP (Nucleocapsid protein; 53.4 kDa), P (Phosphate protein; 41.9 kDa), and M (Matrix protein; 39.8 kDa) based on their predicted molecular weights [50, 51]. **f** Transmission electron micrographs of negatively stained feed and elution fractions. Samples were adsorbed to 200 mesh formvar–carbon copper grids, negatively stained with 2% uranyl acetate and viewed on a FEI Tecnai G2 F20 at 200 kV scanning transmission electron microscope. The fuzzy surface suggests the presence of a glycoprotein layer on the outside of the virion. Scale bars denote 100 nm (left panel), 50 nm (center panel), and 20 nm (right panel). Images were recorded at magnifications of 50,000x (left panel), 160,000x (center panel), and 350,000x (right panel)

To evaluate the reproducibility of virus purification using the larger size NatriFlo® HD-Q membrane, three independent experiments were conducted, each with a different 200 mL batches of feed with starting titers of 2.38 × 10^7^, 3.× 10^7^ and 7.50 × 10^7^ TCID_50_/mL. The amount of virus, protein, and DNA found in the feed, flow through, washes and elutions was quantified and the results summarized in Table 1. As much as, 1.67 × 10^10^ TCID50/mL of virus was loaded onto the membrane and in each purification run, breakthrough was not achieved. On average, 84% + 10% of the virus bound the NatriFlo® HD-Q membrane and 71% + 10% of the virus was recovered in the elution (Fig. 7a and b). In the presence of 100 mM citric acid as an interference agent, an average of 74% + 5% of total protein did not bind the membrane and was found in the flow through (Fig. 7c), thereby allowing preferential binding of the virus to the membrane. In the elution there was a carryover of only 3% + 2% of total protein. Quantification of DNA using the Qubit dsDNA HS assay revealed a carryover of 30% ± 16% DNA in the elution (Fig. 7d). To confirm purity, 10 μL of the feed, flow through, washes and elutions were separated by sodium dodecyl sulfate polyacrylamide gel electrophoresis (SDS PAGE) and stained with Coomassie brilliant blue (Fig. 7e). The feed and flow through fractions contained a significant amount of protein that was not detected in elution fractions 1 and 2, which were enriched for five of the major structural proteins of NDV (HN, F, M, NP and P). The L protein, which is produced at the lowest concentration, was not visible. Additionally, the purity and quality of NDV was analyzed by transmission electron microscopy after negative staining (Fig. 7f). An abundance of intact virions was observed in the feed and elution fractions, both containing pleiomorphic shaped virions of a similar size. In comparison to the feed, the elution fraction appeared to contain fewer host cell contaminants and the virus was often found to be aggregated.

**Table 1:** Ions formed at pH 8 for several interference agents (bicarbonate, citrate, EDTA, and phosphate)

Buffer Species	pKa	Ion Form at pH 8
Bicarbonate	6.40, 10.30	1^−^
Phosphate	2.10, 7.20, 12.30	2^−^
EDTA	2.00, 2.70, 6.20, 10.31	3^−^
Citrate	3.13, 4.76, 6.40	3^−^

### Mouse toxicity study

To confirm that NDV purified using the NatriFlo® HD-Q membrane and interference chromatography can be safely administered to mice, a low dose of NDV (10^6^ TCID_50_/mL) and a high dose of NDV (10^8^ TCID_50_/mL) were delivered intravenously to 8-week-old BALB/c mice. Mice were monitored for adverse events, including excessive weight loss (> 20%), ruffled coat, hunched appearance, apathy, and respiratory distress daily for 14 days post-administration. No adjustment (other than dilution for the low dose using phosphate buffered saline) was made to the chromatography elution fraction prior to injection. At the completion of the study the mice given the low dose of NDV (10^6^ TCID_50_/mL) and the mice given the high dose of NDV (10^8^ TCID_50_/mL) had no significant changes in their biochemistry, complete blood count, or clinical features (data not shown). The toxicity study results were the same as for mock infected mice indicating that the purified NDV is safe for injection into mice.

## Discussion

### Proposed mechanism of interference chromatography

The effectiveness of chromatography is influenced by the buffer conditions and the molecular, ionic diversity of the matrix in which a target molecule resides in solution. The interference chromatography concept uses molecular interactions between the mobile phase matrix, the target molecules and impurities, and the chromatographic solid phase media. This interference method allows for improvements in performance of any chromatographic approach, such as superior resolution, improved binding capacity or augmented flow through productivity. Variations of this technique with anion exchange resins or membranes have been successfully used in the past for virus purification of Influenza H1N1 virus [52] and Influenza A/B virus [53]. One study showed that anions from triprotic kosmostropes, phosphate and citrate, with anion-exchange resins having quaternary amine or primary amine functional groups showed good virus binding, with improved binding by the salt-tolerant primary amine in phosphate buffer at pH 8.0 [52]. The other study used a continuous two-column approach with the first column of big bead AEX resin and the second one with a primary amine membrane adsorber with multivalent ions and both chromatography steps run in the negative-binding mode (also known as Flow Through Chromatography) to purify Influenza virus [53]. The study demonstrated the reduction of host DNA on the membrane was very impressive with complete recovery of virus [53].

In this study, a new advanced technology hydrogel chromatography membrane (NatriFlo® HD-Q) was used to demonstrate a single-step NDV virus purification in positive-binding mode using interference agents prior to (sample treatment) and throughout the chromatography. The hydrogel chromatography membrane is a very high density ligand membrane that contains quaternary amine functional groups at 925 ueq/mL and binds 240 mg/mL BSA at a dynamic 10% breakthrough binding capacity at 10 membrane volumes/minute or 6 s of residence time (data not shown). Therefore, the ability of this new technology chromatography membrane to bind virus at resin capacity at membrane flow rates and using interference multivalent ions was evaluated and exploited for a one-step NDV purification and at constant pH of 8.2. The interference chromatography approach creates interference in the sample (for this study, it the interactions were mainly charge shielding and van der Waals forces, similar to methodologies used previously [52, 53] and the equilibration buffer in order to influence separation between biomolecules in the matrix, i.e. product-related and process-related molecules. Prior to chromatography, interference agents are added to the virus sample to improve the separation capability during the chromatographic process with the solid phase matrix, so there are stronger molecular and ionic interactions in every microenvironment solution-solid phase chemistry. For the solid phase anion exchange hydrogel matrix of HD-Q in this study, the high density of ligands creates salt tolerance by multi-site binding with target molecules/impurities, so multivalent ions, such as phosphate, citrate, and EDTA can have a displacement effect on the solid phase for weaker binding molecules, like host cell proteins. Specifically, the sample and mobile phase matrix conditions were altered by addition of molecule effectors, that included bicarbonate, phosphate, EDTA, and citrate, to induce molecular interactions and improved separation during the chromatography operation.

Establishment of microenvironments, somewhat like theoretical plates, can effectively separate the target molecule from a complex milieu. Depending on the chromatographic mode, the target molecule can flow through the media while impurities bind (negative mode [53];) or the target molecule is bound to the chromatographic support and the impurities pass through. The latter option, often referred to as bind-and-elute mode ([52]; and the current study), then requires an elution step to remove the target molecule from the solid phase. In this study with the binding of NDV to the HD-Q hydrogel matrix, the host cell proteins flowed through the chromatography membrane and then the NDV was reverse eluted in strong salt (1 M NaCl) conditions to recover the virus. A downside to typical ion exchange separations is that species with a charge distribution similar to the target molecule will interact with the charged media in the same manner as the target, therefore resulting in some impurities still present in the process stream after the chromatography purification step. This is true for both chromatography forms, flow through and bind-and-elute mode. The pre-established microenvironments enhance separation of closely-related species by adding a separation dimension prior to chromatography (for ionic environments as evaluated in this study, the charge shielding can help further separate those closely-related species and this established solution condition improves chromatography separation when the material is introduced to the solid phase ion exchange material). This allows for improved separation of molecules with overlapping chromatography profiles and a magnification of separation power, so there is an increase in the number of closely-related, overlapping molecules able to separate from each other with an increase in resolution.

**Fig. 8:**
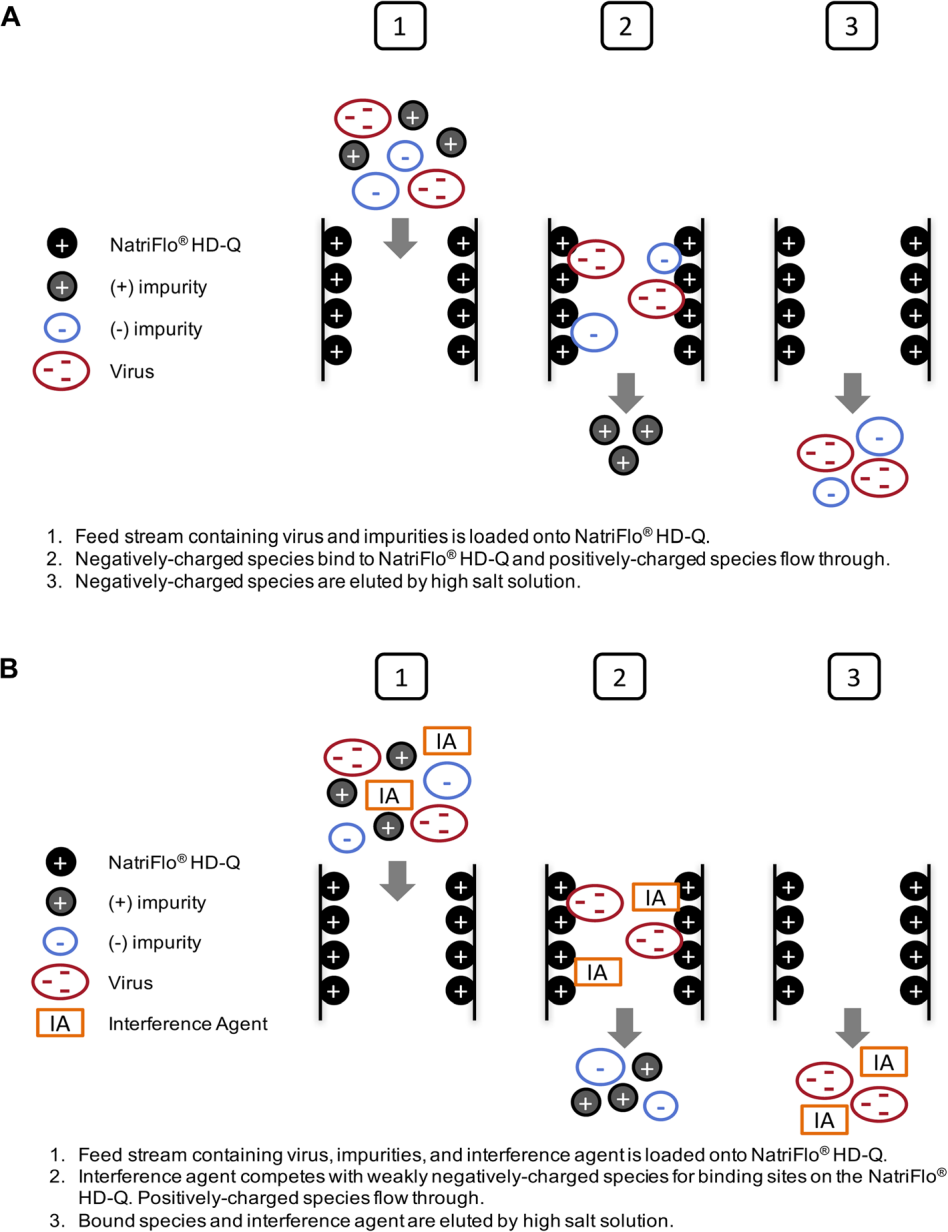
Illustration of anion exchange chromatography using bind-and-elute mode for virus purification. **a** Conventional operation of anion exchange chromatography for virus purification. **b** Proposed mechanism of interference chromatography in which an interference agent (citrate) reduces the binding of impurities, therefore improving the purity of virus

Anion exchange (AEX) chromatography is often used in the bind-and-elute mode for virus or viral particle separations because they are typically negatively-charged at physiological pH [54]. Figure 8 depicts virus purification using AEX chromatography in bind-and-elute mode. In typical AEX operation (Fig. 8a), different species of similar charge bind to the stationary phase with the target protein, which leads to impurities in the elution. For interference chromatography (Fig. 8b), it is proposed that purity is improved by adding an interfering agent that competes with other molecules of similar charge and interacts with the chromatography media, therefore inhibiting binding of other weakly negatively-charged molecules.

### Selection of interference agent and its concentration

Selecting appropriate interference agents and their concentration requires careful examination of the agents’ effect on impurity removal and virus recovery. Certain agents such as citrate and EDTA create stronger interference compared to others like bicarbonate and phosphate, as illustrated in Fig. 1. A possible explanation for these different behaviors is the difference in the valence of the ions and the ion forms present at the loading pH, shown in Table 2. In the case of interference chromatography with NDV, more negatively-charged interference agents can induce a stronger weakening effect on the interactions between the HD-Q membrane and total protein, leading to better impurity clearance. Since each family of virus will require a specific condition to preserve stability and infectivity, selection of the appropriate interference agent and its concentration needs to be evaluated on an individual basis. In the case with this NDV purification work, 100 mM citrate was selected as citrate provides better impurity removal than phosphate and more effective infectivity preservation than EDTA.

**Table 2:** Analysis of large scale purification runs

	Total			% of Total		
	Infectious Particles (TCID_**50**_)^**b**^	Protein (ug)	DNA (ug)	Infectious Particles (TCID_**50**_)^**b**^	Protein (ug)	DNA (ug)
**Run #1**						
**Feed**	1.67E+ 10	404.9	22.95	100%	100%	100%
**Flow Through**	2.04E+ 09	164.4	14.11	12.2%	64%	62%
**Wash 1**	3.36E+ 08	0^a^	0.50	2.0%	0.0%	2.2%
**Wash 2**	8.00E+ 06	0^a^	0^a^	0.05%	0.0%	0^a^
**Elution 1 (20 mL)**	1.05E+ 10	19.1	8.95	63	4.7%	39%
**Elution 2 (20 mL)**	9.38E+ 08	18.6	0.69	6%	4.6%	3%
**Run #2**						
**Feed**	5.58E+ 09	504.3	21.16	100%	100%	100%
**Flow Through**	3.14E+ 08	349.7	12.73	6%	69%	60.17%
**Wash 1**	1.25E+ 07	25.55	0.37	0.22%	5%	1.77%
**Wash 2**	1.91E+ 06	21.1	0^a^	0.03%	4.2%	0.00%
**Elution 1 (20 mL)**	3.56E+ 09	12.7	8.43	64%	2.5%	39.83%
**Elution 2 (20 mL)**	2.42E+ 08	10.1	0.35	4%	2.0%	1.66%
**Run #3**						
**Feed**	4.84E+ 09	610.7	17.028	100%	100%	100%
**Flow Through**	9.14E+ 08	482.3	15.114	18.88%	75.91%	88.8%
**Wash 1**	2.10E+ 08	46.7	0.534	4.34%	7.65%	3.1%
**Wash 2**	1.19E+ 06	36.6	0^a^	0.02%	6.00%	0^a^%
**Elution 1 (20 mL)**	3.61E+ 09	22.2	1.91	74.64%	3.64%	11.2%
**Elution 2 (20 mL)**	1.21E+ 08	24.3	0.237	2.49%	3.98%	1.4%

^a^Below level of detection

^b^Number of infectious particles were determined in triplicate using the TCID_50_ method

### Evaluation of process productivity

In opposition to vaccines that require small doses of viral antigen, oncolytic virotherapy requires much higher doses of pure viruses to induce the direct and immune-mediated mechanisms to destroy malignant cells. However, low productivity has been a key issue the industry is facing for manufacturing clinical-grade OVs as most currently used purification architecture involves many filtration, concentration, and centrifugation/ultracentrifugation unit operations in order to achieve the target purity requirement [55]. Unit operations such as ultracentrifugation are time- and labor-intensive and difficult to scale up for clinical manufacturing. Moreover, complex purification trains are unfavorable from a productivity point of view due to extended processing time and low yields. For NDV purification discussed in this paper, a significant gain in productivity was observed, even over the most recent methods [56], by replacing the commonly used TFF and ultracentrifugation-based purification scheme with high throughput membrane chromatography operated in the presence of interference agents we have increased average virus recovery from ~ 60% to 71. The processing time was reduced from 2 days to less than 1 hour. Moreover, thanks to the better overall recovery in the membrane process (90–100% versus 60–65%), upstream production (i.e. production in eggs) can be downsized. With the current loading capacity, one run on a commercially available membrane column with 460 mL membrane volume will be sufficient to support 3–5 treatments of a Phase I study [41].

On average, 71% of the virus was eluted, leaving approximately 14% of bound virus unaccounted for. Loss of infectious virus could be due to a variety of reasons including changes in pH which inactivate the virus, strong adherence of the virus to the membrane, or shearing of the virus due to pressure build up. Additionally, it is possible that virus aggregation may have led to an underestimation of the true titer. While a significant amount of work was done to recover as much NDV as possible, there are other options that can be explored to further optimize this process.

We observed larger differences in DNA removal for the trial runs, in which three different sizes of membranes were used. This could potentially be due to batch to batch variability in the feed or differences in the membranes. When we scaled up production and used the same sized membrane, we observed less variability in DNA removal (70 ± 16% DNA).

### Future perspectives of interference chromatography

In the case of NDV purification, further studies can be done to improve upon the process that has been described. It is proposed that adding an interference agent to the elution would enable a reduction in the strength of the elution species needed to remove the target from the chromatography media (which is synonymous with displacement chromatography). The current elution condition requires high NaCl concentration (minimum 1 M) to achieve good virus recovery (see Figure 1S in Supplemental Data). Mild elution conditions are beneficial for labile viruses and proteins and may enable elution collection that can be used directly for therapeutic use, simplifying or eliminating a buffer exchange step before formulation. Exploring various buffer conditions may also improve the DNA removal. The optimal elution condition would completely elute the virus, but leave the DNA bound to the membrane which can subsequently be disposed of if a single-use strategy is implemented. In a review by Ungerechts et al., 2016 [20], which provides an overview of clinical-grade manufacturing procedures for OVs, nuclease treatment to degrade host cell nucleic acid prior to chromatography was common to all OV purification processes, and thus would likely be implemented prior to purification of clinical grade NDV using interference chromatography.

A challenge with interference chromatography is that the process development method requires investigation, characterization, and may not conform to standard process platforms. The method utilizes knowledge of the sample matrix, products/variants, and impurity levels and requires an empirical study for optimum separation and purity. This methodology necessitates increased development times and costs due to extensive design of experiment (DOE) testing. It is largely unknown how interference agents will interact with different target species such as viruses, virus-like-particles (VLPs), antibodies, or proteins. NDV used as a model, achieved single-step purification of proteins and dramatically reduced processing time, but this is not necessarily true for all targets. Interference chromatography may need to be complemented with other polishing methods (for example, ultracentrifugation to resolve empty from full virus particles). Moreover, further steps would be needed in order to generate clinical grade virus. The virus would have to be subjected to dialysis or diafiltration to exchange it into a more physiologically balanced buffer and electrolyte solution as well as to improve storage.

Once established, however, the interference technique should be very reproducible and could separate more difficult molecules, like viruses or labile proteins where standard chromatographic methods do not perform adequately. The interference chromatography method is expected to be easily scalable since the interference agent concentration is independent of the purification scale. Depending on the chromatography media, the consumables and processing equipment can also be easily scaled up to increase purification efficiency. It is anticipated that interference chromatography can be applied in either positive or negative chromatography modes. Currently traditional IEX chromatography is often used in the manufacturing of several vaccines including hepatitis A (EP 0538142 A2, 1994), hepatitis B (WO 02/122887 A1, 2002), polio [57], adenoviral vectors [58], Ebola virus glycoproteins [59], and influenza VLPs [60]. Introducing interference methods to these AEX processes may produce purer product in less DSP steps and higher yields and therefore lower production costs.

## Conclusion

The interference chromatography method is a powerful chromatography method for the separation of impurities from product molecules and the separation of closely-related product variants. This chromatographic method establishes an in-solution gradient of molecule interactions using simple interfering agents that enhance or inhibit in-solution matrix interactions with the target product. The combination of interfering agents and chromatographic separation actually mimics a quasi, multi-dimensional chromatographic separation, but in a single chromatography step. The impact of the interference method is a more powerful (stronger interactions of targeted product molecules or removal of unwanted impurities), combinatorial chromatography operation that increases separation efficiency and simplifies downstream processing with fewer steps. This provides benefits in purification speed and yield that enables cost effective therapeutics to be delivered to the patient faster. It was demonstrated here that NDV was able to be purified from allantoic fluid in a single-step using interference agent, multi-valent citrate, and HD-Q membrane chromatography, which reduced protein by > 97% and DNA up to 70% with good recovery of virus > 70%.

## Methods

### Cell culture

DF-1 chicken embryo fibroblast cells (obtained from the American Type Culture Collection; ATCC® CRL-12203) were maintained in Dulbecco’s modified Eagle’s medium, supplemented with 10% bovine calf serum (BCS), 2 mM L-glutamine, 100 units/mL penicillin and 100 μg/mL streptomycin at 37 °C in 5% CO_2_/air atmosphere.

### Virus production

The NDV-F3aa-GFP genome and helper plasmids were purified with the GenElute HP Plasmid Maxiprep Kit. Recombinant NDV-F3aa-GFP was rescued and propagated in specific pathogen free eggs as described [56]. Allantoic fluid was harvested at 50 h post-inoculation and clarified by centrifugation (1,500 x *g* for 10 min at 4 °C). To confirm the presence of NDV in the allantoic fluid, a hemagglutination assay (HA) was performed as described [56]. The average virus titer of pooled allantoic fluid was 1 × 10^8^ TCID_50_/mL. Virus was stored at − 80 °C.

### Chromatography

For all experiments, virus containing allantoic fluid was thawed at 4 °C overnight, equilibrated to room temperature, and clarified by centrifugation (1,500 x *g* for 10 min) followed by the addition of 60% sucrose to a final concentration of 2.5%. It is important to note that using cold feed (i.e. 4 °C) can cause a spike in pressure, potentially due to aggregate formation, and may impede loading of the virus onto the membrane and subsequent elution. Therefore, it is recommended that once the virus feed is supplemented with interference agent and sucrose, it be allowed to reach room temperature before 0.45 μm filtration and left at room temperature for the duration of the run. For screening tests, an appropriate volume of concentrated interfering salt solution [1 M monobasic sodium phosphate, 1 M citric acid, 0.77 M sodium bicarbonate, or 0.24 M ethylenediaminetetraacetic acid (EDTA)] was added to harvested allantoic fluid to achieve the desired interfering agent concentration (20 mM, 40 mM, 60 mM, 80 mM or 100 mM). For control tests, there was no adjustment to allantoic fluid besides the addition of sucrose. For salt comparison tests, the conductivity of allantoic fluid was adjusted with NaCl to 25 ms/cm, to normalize all samples to the conductivity of the feed with 100 mM citrate. All feeds were filtered with a 0.45 μm PES bottle top filter. All experiments were performed with 1 layer of NatriFlo® HD-Q membrane (membrane volume = 0.1 mL) assembled in a 25 mm diameter stainless steel housing (25 mm SS device). All experiments were performed on a KDS 220 Multi-Syringe Infusion Pump with a flow rate of 20 membrane volumes (MV) per minute. Pressure was kept under 15 psi, as NDV is known to be sensitive to shearing at high pressure. The membrane was first equilibrated with equilibration buffer (25 mM Tris with appropriate interfering agent concentration, pH 8.2) for 5 mL. After sample loading (5 mL for screening, control, and NaCl conductivity control, and 13 mL to 37 mL for capacity test) the membrane washed with 5 mL of equilibration buffer followed by a second wash with low salt buffer (5 mL of 25 mM Tris, 100 mM NaCl, pH 8.2). The flow direction was then reversed for elution to reduce shearing and ensure good recovery. Step elution conditions (25 mM Tris with 0.5 M NaCl, 1 M NaCl, 1.5 M NaCl, 2 M NaCl, and 2.5 M NaCl, pH 8.2) were used for all screening tests as well as control and salt comparison tests. One-step elution with 25 mM Tris, 1 M NaCl, pH 8.2 was used for loading capacity test. This elution was chosen because it was the optimal balance between NDV recovery and reduced NaCl concentration (see supplementary Figure S1 for further details).

### Scale up

Scale up was performed as described above with some minor changes. Briefly, the interference agent 100 mM citric acid (pH 8.2) was added as a 10x buffer to the feed (equilibrated to room temperature), followed by the addition of 60% sucrose to achieve a final concentration of 2.5% sucrose. The feed was then 0.45 μm filtered immediately prior to being loaded onto the membrane. All experiments were performed with a Masterflex L/S peristaltic pump (Cole Parmer, USA) and Masterflex L/S 14 BioPharm Plus Platinum-Cured Silicone Pump tubing. Membranes were first equilibrated with 40 mL of equilibration buffer (25 mM Tris with 100 mM citrate as interfering agent, pH 8.2). Once the feed was loaded onto the membrane, this was followed by wash 1 with 40 mL of equilibration buffer and wash 2 with 40 mL of low salt buffer (25 mM Tris, 100 mM NaCl, pH 8.2). The flow direction was then reversed for elution to reduce shearing and to ensure good recovery. Elution was performed using 40 mL of 25 mM Tris, 1.0 M NaCl buffer, in two 20 mL fractions.

### Total protein quantification

Total protein was quantified in triplicate using the Bio-Rad Protein Assay kit (Bio-Rad Laboratories), which is based on the method of Bradford, in a flat bottom 96-well microtiter plate according to the manufacturer’s instructions. Bovine serum albumin (BSA) (New England Biolabs) was used to generate a standard curve, which ranged from 0 to 20 μg/mL BSA.

### Characterization of virus in the elution solution

Samples (3 μL) of the feed and elutions from the three large scale purification experiments were adsorbed to 200 mesh formvar–carbon copper grids for 2 min at room temperature and excess liquid was removed. The grids were then negatively stained with 2% uranyl acetate and viewed on a FEI Tecnai G2 F20 at 200 kV scanning transmission electron microscope. Images were recorded at a magnification of 50, 000X, 160,000X and 350,000X with a Gatan Ultrascan 4 k × 4 k CCD camera (Gatan, Pleasanton, CA, USA) and all data processing and analysis was performed using the Gatan Digital Micrograph software (Molecular and Cellular Imaging Facility at the University of Guelph).

Sodium dodecyl sulfate polyacrylamide gel electrophoresis (SDS-PAGE) was performed to visualize protein content in the feed, flow through, wash and elution from the three large scale purification experiments. Briefly, 10 μL of feed, flow through, wash 1, wash 2 and elutions 1–5 were denatured at 100 °C for 5 min in 1× SDS PAGE sample buffer containing 143 mM β-Mercaptoethanol, separated by SDS–PAGE on 12% Tris–glycine gels and stained with Coomassie Blue R250.

### TCID_50_ assay

NDV-GFP titer was determined by 50% tissue culture infective dose (TCID_50_) assay and expressed as TCID_50_/mL. NDV samples were serially diluted 10-fold with 1x Dulbecco’s phosphate-buffered saline (DPBS) from 10^− 1^ up to 10^− 8^ and 10 μL were added to DF-1 chicken fibroblast cells grown in DMEM (supplemented with 4 mM L-glutamine, 7% bovine calf serum) seeded at 70–80% confluency in a 96-well cell culture plate (*n* = 8). After 96 h, green fluorescence was observed using a Carl Zeiss Axio® 154 Observer A1 inverted fluorescence microscope and titer was calculated according to the Spearman-Kärber method (Ramakrishnan, 2016) [61].

### Total DNA quantification

Total DNA from feed, flow through and elution fractions were purified using Qiagen DNeasy Blood & Tissue Kit, including the RNase A (Invitrogen, Canada) step, according to the manufacturer’s protocol. Fluorometric quantification of DNA was performed using a high sensitivity dsDNA Qubit® assay using a Qubit® 2.0 Fluorometer (Genomics Facility, Advanced Analysis Center, The University of Guelph, Canada).

### Toxicity testing

Mouse toxicity experiments were performed in compliance with the guidelines set forth by the Canadian Council on Animal Care. The Animal Care Committee at the University of Guelph approved all methods. Randomly allocated groups of six eight-week old female BALB/c mice purchased from Charles River Laboratories (Wilmington, MA) were housed at the University of Guelph in a specific pathogen-free isolation facility. Mice were housed in groups of four and food (Teklad Global 14% Protein Rodent Maintenance Diet, Indianapolis, USA) and water (tap) were provided ad libitum. Mice were acclimated to the environment for 7 days prior to study initiation. Mice received 100 μl injections intravenously of 1 M NaCl (control), 1 × 10^6^ TCID_50_ units of purified NDV in 1 M NaCl (low-dose) or 1 × 10^8^ TCID_50_ units of purified NDV in 1 M NaCl (high dose). Mice were observed for changes in body weight or behavior over a 2-week period. On day 14 mice were euthanized by anesthetizing with isoflurane prior to cervical dislocation and blood was drawn for complete blood count and biochemical analysis, performed at the Animal Health Laboratory, the University of Guelph.

## Supplementary information

**Additional file 1.**

**Additional file 2.**

## Data Availability

All data are included with the manuscript. All data generated or analyzed during this study are included in this published article and its supplementary and additional information files.

## Abbreviations

TFF: Tangential flow filtration
OVs: Oncolytic viruses
IEX: Ion exchange
NDV: Newcastle disease virus
HCP: Host cell protein
LRV: Log reduction value
MV: Membrane volume
AEX: Anion exchange
DOE: Design of experiment
VLP: Virus like particle
BSA: Bovine serum albumin
EDTA: Ethylenediaminetetraacetic acid
