# Supplementary figures and images for: Interference chromatography: a novel approach to optimizing chromatographic selectivity and separation performance for virus purification
Source: BMC Biotechnol. 2020 Jun 17;20:32. doi: 10.1186/s12896-020-00627-w (PMC7301511; doi:10.1186/s12896-020-00627-w)

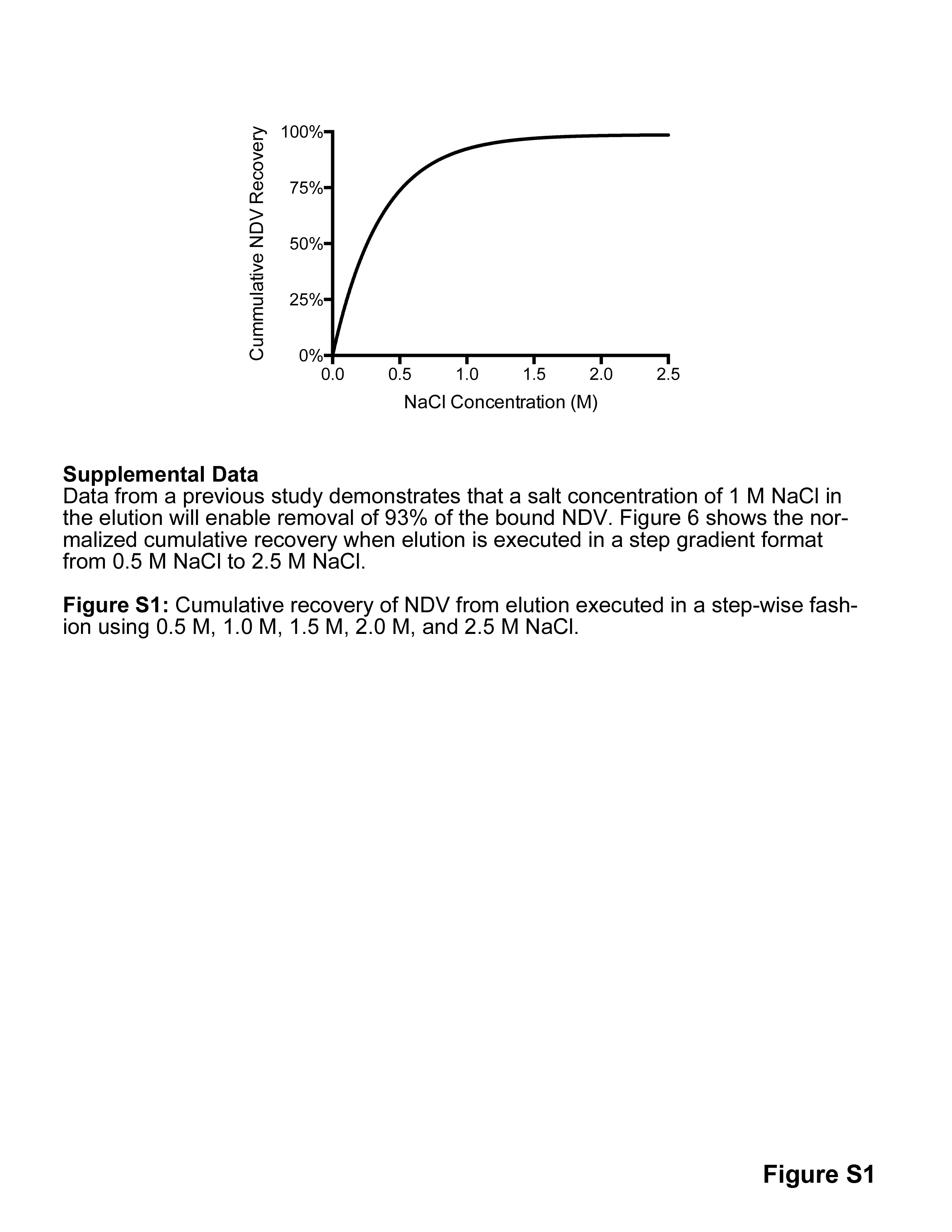

Supplement: Supplementary file 1 — Additional file 1. [file 12896_2020_627_MOESM1_ESM.tif]
